# Characterization of Two Cactus Formulation-Based Flocculants and Investigation on Their Flocculating Ability for Cationic and Anionic Dyes Removal

**DOI:** 10.3390/polym12091964

**Published:** 2020-08-30

**Authors:** Bouthaina Othmani, José A. F. Gamelas, Maria Graça Rasteiro, Moncef Khadhraoui

**Affiliations:** 1 Laboratory for Environmental Engineering and Eco-technology, ENIS, University of Sfax, Sfax 3038, Tunisia; bouthainaothmani91@gmail.com; 2 Department of Chemical Engineering, CIEPQPF, FCTUC, University of Coimbra, 3030-790 Coimbra, Portugal; jafgas@eq.uc.pt

**Keywords:** coagulation-flocculation, methylene blue, methyl orange, oven-dried cactus powder, lyophilized powder, polyacrylamide

## Abstract

Dye invasion in wastewaters is undeniably one of the crucial environmental concerns in addition to the supplement of toxic synthetic chemical flocculants used for color removal using the conventional coagulation-flocculation process. With the aim to improve the flocculation stage in terms of reagents safety and ensure dyes removal, the present study explores the flocculating effectiveness of two natural, stable, and eco-friendly cactus formulations, namely 60 °C oven-dried (DP) and lyophilized (LP) cladodes. Both formulations were assessed to treat cationic (Methylene blue; MB) and anionic (Methyl Orange; MO) dye solutions as a substitution attempt for the currently questioned employed synthetic chemical flocculants. Obtained results demonstrate that, in conjunction with alum as coagulant, the lyophilized powder (LP) bio-based flocculant appears to be the most efficient cactus formulation, showing a significant color (83%) and a turbidity (69%) abatement for the cationic dye (MB) and, respectively, 63% and 62% for the anionic one (MO). Additionally, the flocculation activity of the LP formula remained high over an eight-month period of storage. Moreover, based on the Fourier transform infrared (FTIR) spectroscopic analysis and the chemical characterization of cactus formulations, the occurring flocculation mechanisms of the dye removal are presumed to be based on both adsorption and bridging phenomena. Further, the significant color and turbidity decline achieved upon the addition of the lyophilized cactus cladodes powder (LP), enhancing thus the coagulation performance of the alum-based coagulant, proved the effectiveness of this bio-flocculant compared to the commonly used chemical flocculant (polyacrylamide). Hence, it was suggested that lyophilized cactus cladodes as a natural flocculant could be one of the effective surrogates to chemical flocculants conventionally used in wastewater treatment for the sake of a safer and sustainable environment.

## 1. Introduction

Nowadays, a variety of dyes are being extensively exploited in many industrial fields and it is broadcasted that more than 7 × 10^5^ tonnes of commercial dyes were produced annually to fulfill the manufacturing requirements [1]. They have been used in plastic, cosmetic, pulp and paper industries, tannery, and mostly textile industries where, for instance, around 100,000 different kinds of dyes have been utilized [2].

Based on the dye molecule surface charge, dyes are labeled cationic such as the azo dyes (Methylene Blue), anthraquinone dyes (Purpurin), anionic dyes (Methyl Orange), reactive dyes (Alizarin Yellow), and non-ionic dyes which are mainly disperse dyes (Disperse Blue 79). Among the aforementioned dyes, methylene blue and methyl orange subsist usually in water streams due to their massive usage causing thus serious environmental threats [3]. Their irresponsible and incessant dumping into receiving water bodies is claimed to cause a burden to the environment and human health [4]. These environmental threats range from smell and taste alterations in water sources [5], abstraction of sunlight in aquatic medium resulting subsequently in dissolved oxygen fall, to their low bio-degradability tendency or their partial degradation giving rise to new intermediates products such as aromatic amines, azo or sulfur compounds which are more toxic than the original mother molecules [6]. Bearing in mind the acute environmental and health risks, dye molecules have to be removed before being poured into receptor media.

To do so, various technologies have been developed for dye effluents treatment. For instance, adsorption [7], anaerobic treatment [8], filtration [9], advanced oxidation processes [10], and coagulation-flocculation [11] were evaluated in several former studies.

Among the aforementioned techniques, the coagulation-flocculation remains one of the most extensively used process owing to its simplicity, rapidity, low operating and maintenance cost [12]. Moreover, it ensures a direct dye settlement without decomposition into toxic intermediates [13]. However, despite the effectiveness of this well established and widespread method, the used flocculants (organic polymers: polyacrylamide or acrylic acid) therein are questioned for their deleterious effects on the environment [14] and human health [15] in addition to the residual monomers that usually remain in the supplied products. Additionally, a notable organic residue may emerge in the treated water [16], generating subsequently toxic and low bio-degradable sludge [17].

In fact, depending on water pollutant characteristics, addition of chemical flocculants upon the coagulation step (using metal salts), is quite of importance to ensure an effective pollutant separation from the treated water. However, in spite of their efficacy, the global awareness about their environmental effects, is considered one of the motivating points underlying the search for more environmentally-friendly classes of flocculants for water treatment. Therefore, a supplement or a total substitution of these synthetic flocculants by safe and natural ones is worthy to be investigated.

In-line with this objective, bio-coagulants and bio-flocculants derived from animals (Chitosan) [18] or plant extracts (wood, shells, seeds or leaves …) [19] have been receiving a great interest as promising alternatives to chemical flocculants owing to their harmless, natural abundance, cost-effectiveness and bio-degradability [20]. So far, the promising lab results found using these natural products are the driving force behind exploring more bio-based coagulants and flocculants.

Among the so many studied bio-based materials, cactus cladodes, commonly known as *Opuntia ficus indica* are well recognized by their coagulation-flocculation effectiveness in treating various wastewaters [21]. Within this frame, several researchers have investigated the coagulation/flocculation performance of cactus toward natural waters [22], synthetic waters [23] and industrial wastewaters [24]. For instance, in a recent review conducted by Othmani et al. [25] disclosing some promising results using plant based-flocculants, it was pointed out the efficacy and ability of cactus to behave as a sustainable and eco-friendly coagulant and flocculant toward a wide range of pollutants. Besides, Kazi and Virupakshi [26] reported outstanding pollutant removal from a tannery wastewater using cactus as coagulant. Additionally, in a survey elaborated by Belbahloul et al. [27], this bio-material was used as flocculant in the treatment of a synthetic turbid water. Therefore, this dual functionality confers to cactus a great interest in water treatment and can be thought to be applied in a point-of-use water treatment technology in developing countries averting, hence, the procurement charge and the detrimental effects encountered with the utilization of its chemical counterparts.

Yet, researchers are still looking for the best formulation that will be further processed and applied at the industrial level. In fact, most of the former studies were dealing with raw cactus juice as bio-flocculant, despite its questionable shelf life due to the presence of proteins and carbohydrates (active components) in an aqueous medium and which are subjected to denaturation and alteration. We recall that it is well known that high humidity induces the growth of pathogenic fungi and bacteria that cause spoilage and degradation in cactus mucilage quality. Producing a stable and water-free cactus bio-based flocculant formula that can convince wastewaters’ practitioners is another challenging task for researchers.

In Tunisia, since cactus plants are so abundant, the valorization of this bio-resource and looking for a more stable flocculant formula with a longer shelf life than the previously tested cladodes raw juice prescription [28] is, therefore, worth to be explored. Thus, the present study aims to investigate the flocculation ability of two powdered cactus cladodes formulations; (i) an oven-dried at 60 °C and (ii) a lyophilized one. These two forms were assessed to treat a cationic (Methylene Blue (MB)) and an anionic (Methyl Orange (MO)) dye solutions in combination with alum as coagulant. Further, both products were subjected to a bio-chemical characterization and FTIR spectroscopy measurement to get ideas concerning the possible components responsible for dyes removal and to elucidate the plausible flocculation mechanism. Dyes removal was evaluated in terms of color and turbidity drops. To assert the efficiency of cactus powder-based flocculant, a comparison with the most commonly used chemical flocculant (polyacrylamide) was also carried out. The longevity of the best flocculant formula was also assessed over a period of eight months.

## 2. Materials and Methods

### 2.1. Materials

The two dyes; Methylene Blue (MB) and Methyl Orange (MO) were supplied by Sigma-Aldrich, Steinheim, Germany, to simulate synthetic dye solutions. The green cactus cladodes were harvested from a farm located in Sfax region, Tunisia. All chemicals, used on the characterization of the cactus cladodes powders, were analytical reagent grade and purchased from Sigma-Aldrich, Steinheim, Germany. They were applied without any further purification. Aluminum sulfate (Al_2_(SO_4_)_3_ 18H_2_O) was provided by Suvchem, Mumbai, India. The chemical flocculant, non-ionic polyacrylamide, was supplied by Sanfeng Environmental Science Group Co., Ltd., Shandong, China. With reference to the American Public Health Association (APHA) standard method [29], synthetic dyes removal was monitored via pH, absorbance, and turbidity measurements. pH was checked with a pH meter (Inolab 7110, Xylem Analytics, Oberbayern, Germany), absorbance was registered at the wavelength of each dye’s absorbance maximum by a UV-Vis spectrophotometer (UV-2450, Shimadzu, Japan), and turbidity measurement was made using a turbidity meter (WTW-TURB550, Xylem Analytics, Oberbayern, Germany). A BX-1000 FTIR spectrometer (Perkin-Elmer, Überlingen, Germany) was used to register FTIR spectra in the region of 450–4000 cm^−1^. The coagulation-flocculation experiments were conducted using a Floclab jar test apparatus (Prolabo, Le Mans, France).

### 2.2. Preparation of Cactus Formulations

**Figure 1:**
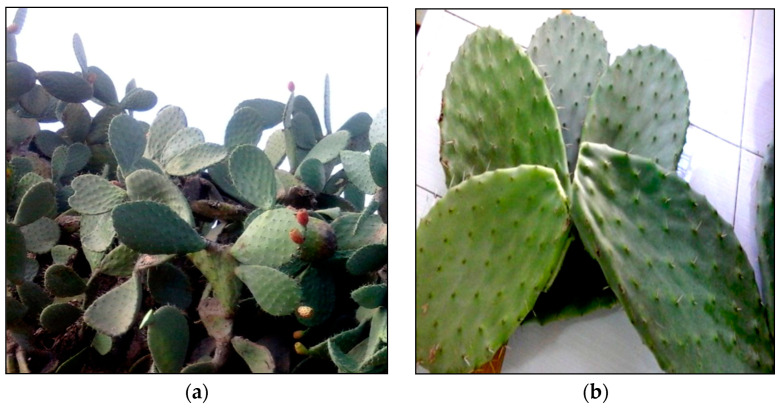
Cactus field (**a**) and the cladodes or pads (**b**).

Mature cactus (*Opuntia ficus indica*) cladodes were collected from Sfax region (Tunisia) in the early autumn (Figure 1). Pads were cleaned from all thorns, repeatedly washed with distilled water and disinfected using sodium hypochlorite to prevent any plausible bacteria growth, then sliced into tiny pieces. To prepare cactus formulations, the small pieces were, either oven-dried at 60 °C for 24 h and then powdered to a sample named DP, or freeze-dried for 24 h in a lyophilizer (P = 130·× 10^−3^ mbar, T = −46 °C) to produce a powder (labeled LP). To assure their separation from the emerging impurities after the drying process such as glochids or fibers as well as to maintain a narrower particle size distribution, both powdered formulations were sieved to 63 µm particle size, hermitically sealed in plastic containers and stored in a desiccator at room temperature for later uses. Effects of storage time on the flocculation performance of the most efficient powder formula were assessed over a period of 8 months. It is worth recalling that the flocculating activity of fresh cactus mucilage is restricted to its shelf life since this material is prone to microbial activity attack due to its considerable water and carbohydrates content [30]. Hence, producing high quality bio-flocculants with longer longevity is one of the driving forces behind exploring the powdered formulations.

### 2.3. Physico-Chemical Characterization of Cactus Formulations

To gain more understanding on their chemical composition and therefore their flocculating ability, determination of the bio-chemical composition of the powdered oven-dried and the lyophilized cactus cladodes was conducted. Based on different standard analytical methods, total phenols, proteins, neutral sugars, uronic acids and klason lignin were quantified. As per the standard reagent, bio-chemicals content was expressed in (mg) equivalent standard reagent per (g) of cactus dry weight. All tests were performed in triplicate for each sample and absorbance records were carried out using a UV-Visible spectrophotometer (UV-2450, Shimadzu, Japan).

Table 1 displays all the experimental procedures followed for the determination of the bio-chemical components of cactus samples.

Further, after incineration and acidic digestion of a known amount of sample, the content of relevant minerals of the cactus powder ashes was determined by atomic absorption spectroscopy (SOLAAR 969) according to the Association of Official Analytical Chemists (AOAC) standard method [36].

Additionally, in order to identify the main functional groups in the powdered cactus formulations, as mentioned above, FTIR spectra were registered by a Perkin Elmer Spectrometer in the region of 450–4000 cm^−1^ using the KBr pellets method.

### 2.4. Preparation of Synthetic Dye Solutions

Along the current investigation, two common dyes were selected: Methylene Blue (MB) and Methyl Orange (MO) which were chosen as models for organic cationic and anionic pollutants, respectively, of dyes manufacturing industries. These two dyes are abundant in wastewater [37] due to their wide application in many industrial fields mostly in textile industry [38]. Synthetic MB and MO stock solutions were prepared by dissolving 0.5 g of each dye in 1 L of distilled water. pH and turbidity were measured according to the APHA standard method [29]. The physico-chemical characteristics of the selected dyes with concentrations of 0.1 g/L, prepared from the stock solution, are exhibited in Table 2.

### 2.5. Coagulation-Flocculation Experiments

The coagulation-flocculation experiments were conducted using a Floclab jar test apparatus equipped with six jars enclosing 500 mL of synthetic dye solution (C = 0.10 g/L) prepared from the stock one by dilution to fit dyes concentration level in wastewater effluent. After a rapid mixing at 150 rpm for 3 min in the presence of 0.30 g/L of aluminum sulfate (Al_2_(SO_4_)_3_·18H_2_O) added as coagulant, a supplement of cactus powder-based flocculant was injected and the mixture was then stirred slowly for 17 min at 60 rpm. After this sequent coagulation-flocculation stage, stirring was stopped. The grouping of alum and cactus powder is believed to be more effective for contaminants removal compared to their independent use [39]. Experimental conditions are chosen based on some preliminary tests and on our previous work [30]. After settling for 30 min, an aliquot of 2 mL from the upper phase of the flocculated solutions was withdrawn for color and turbidity assessment. Flocculation efficiency of the powdered cactus cladodes was evaluated using the following equation:

(1) $$\mathrm{Flocculation}\;\mathrm{efficiency}\;(\%)\;=\;\frac{A-B}{A}\times 100$$

where A and B are the initial and the final absorbance or turbidity of the tested dye solution, respectively.

## 3. Results and Discussion

### 3.1. Bio-Flocculants Characterization

As mentioned earlier, the bio-chemical composition of cactus cladodes was determined to get an idea on the plausible active flocculating agents in the two formulations (LP and DP), and results are listed in Table 3.

The bio-chemical compositions of the two cactus cladodes powders presented in Table 3 show quite comparable concentrations of polyphenols of the order of 2.3 mg/g for LP and 2.0 mg/g for DP. One can notice that though the difference in the powdering process (lyophilizing or oven-drying), both cactus cladodes (LP and DP powders) contain low total phenols load compared to the other bio-chemical components mentioned in Table 3. This result falls in the range of that cited by Aruwa et al. [40] who reported a total phenols content of *Opuntia ficus* cladodes varying from 1.07 to 10.46 mg/g dry weight. Likewise, compared to other plant parts (flower and fruits), Kharrassi et al. [41] found that Moroccan cactus cladodes species hold a polyphenols charge ranging from 3.5 to 12 mg/g dry weight.

Nonetheless, the insignificant difference in the total phenols content, the 60 °C dried pads powder (DP) apparently presents a higher proteins content (64 mg/g) than the lyophilized one (54 mg/g). Within the same line, Kharrassi et al. [41], reported a proteins content in the order of 66.5 mg/g for an oven dried powder of cactus *Opuntia* species. As well, with reference to the lyophilized pads powder, Hernández-Urbiola et al. [42] revealed that, this latter contained 58.5 mg/g of proteins. Table 3 depicts also that both cactus powdered formulations; LP and DP show some amount of klason lignin, in agreement with a previous report where other authors have found 2.5% of lignin in cactus cladodes powder dried at 40 °C for 4 days [43].

As for the mineral composition (Ca, Na, Mg, and Fe), which is comparable for both formulations, a notable calcium concentration, nearly 70 mg/g, was registered for the LP and the DP. This obtained calcium concentration is quite higher than that reported by El-Mostafa et al. [44], which was varying from 5.6 to 18.0 mg/g. However, Bicalho and Penteado [45] claimed that 60% of the mineral composition of cactus pads was calcium. Moreover, Tractenberg and Mayer [46] had reported high deposits of calcium as oxalate crystals in mature cactus pads. As listed in Table 3, calcium is the main mineral present in both powdered cactus formulations, and known to have a crucial role in gelling the pectin fractions. Further, it is believed that this high calcium content may play a prime role in the flocculating behavior of cactus. Indeed, divalent cations mainly Ca^2+^ were reported to neutralize the negative charge carried on the surface of pollutant particles, forming, therefore, bridges between particles and cactus polymer chain [47]. As a result, charge neutralization and bridging mechanisms occurred concurrently, conferring to cactus both a coagulating and a flocculating activity. Moreover, as stated by Wang et al. [48], divalent ions such as Ca^2+^ and Mg^2+^ may contribute to the coagulation activity and would be involved in pollutants removal via double-layer compression.

On the other hand, results depicted in Table 3 indicate obviously that carbohydrates are one of the major constituents of both cactus cladodes formulations. Neutral sugars content was 345 mg/g and 380 mg/g for LP and DP, respectively. The considerable amount of neutral sugars in the DP can be assigned to the temperature effect which may lead to the decomposition of polysaccharides into pentoses and hexoses with reference to Ventura-Aguilar et al. [49] work. It is also worth noting that, based on some other scholars’ works, carbohydrates are assumed to be d-galactose, d-xylose, l-rhamnose, l-arabinose, and galacturonic acid [50].

In contrast, the highest concentration of uronic acids (682 mg/g) was recorded in the LP, exceeding by far the concentration obtained in the DP (546 mg/g). As a comparison, in a study conducted by Rodriguez-Felix and Cantwell [51], it was stated that total carbohydrates (neutral sugars and acids) varied from 640 to 880 mg/g depending on the maturity of cladodes. The relatively low uronic acids content in DP agrees with the rise of neutral sugars concentration in this latter formula upon heating. According to Lefèvre and Tollens [52], the important neutral sugars load is possibly due to the decarboxylation of uronic acids under heating at 60 °C. The same trend was reported by Felkai-Haddache et al. [53] who claimed that the increase of temperature might alter the chemical structure of uronic acids, resulting thereafter a rise in neutral sugars content.

Bearing in mind the significant amount of carbohydrates, the investigated cactus extracts formulations could be an outstanding potential flocculant in water treatment. Furthermore, the paramount uronic acids concentration in the two cactus formulations points to its substantial role in the flocculation activity. Indeed, according to Miller et al. [54], polygalacturonic acid is assumed to be the main flocculating active agent. For better exploring the plausible active agents and functional groups, FTIR spectra of both cactus formulations were recorded.

**Figure 2:**
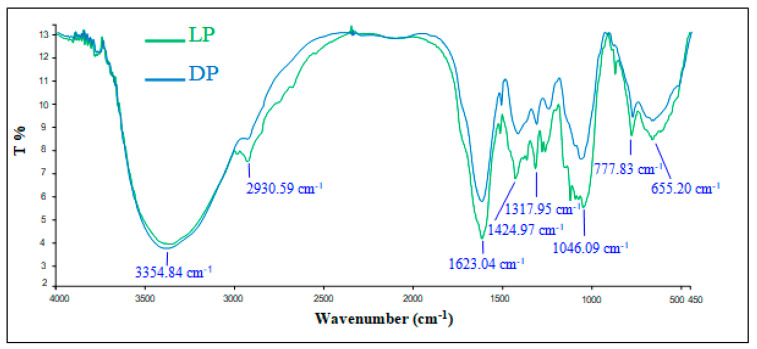
FTIR Spectra of two cactus formulations (LP and DP).

The FTIR spectra presented in Figure 2 provide information about the main functional groups of the two cactus formulations showing similar structures. The recorded bands are mainly: a broad band with a maximum around 3355 cm^−1^ that can be associated to stretching vibrations of hydroxyl (–OH) groups of carbohydrates and N–H groups of glycoproteins with a high degree of intra- and inter-molecular hydrogen bonding; a band at 2931 cm^−1^ attributed to C–H stretching vibration; bands at 1623 and 1425 cm^−1^ attributed, respectively, to asymmetric and symmetric stretching vibrations of ionized carboxylic acid groups (COO^−^), namely from polygalacturonic acid; a shoulder at around 1730 cm^−1^ from C=O stretching of non-ionized COOH groups and substituent methyl ester groups in polygalacturonic acid; a band with maximum at 1046 cm^−1^ mainly due to the C–O stretching of alcohol/ether groups in the carbohydrates [47]. On the other hand, and as presented in Figure 2, it is worthy to point out a slight decrease in the DP peaks intensity in the range of 2930 to 655 cm^−1^ which can be associated to a thermal dehydration effect [43].

As presented in Table 4, the depicted bands in the FTIR spectra can be mainly attributed to polysaccharides, presuming therefore their salient potential in the flocculating activity of cactus pads.

### 3.2. Methylene Blue (MB) and Methyl Orange (MO) Removal Using Cactus Formulations: Bio-Flocculants Optimal Dosage

**Figure 3:**
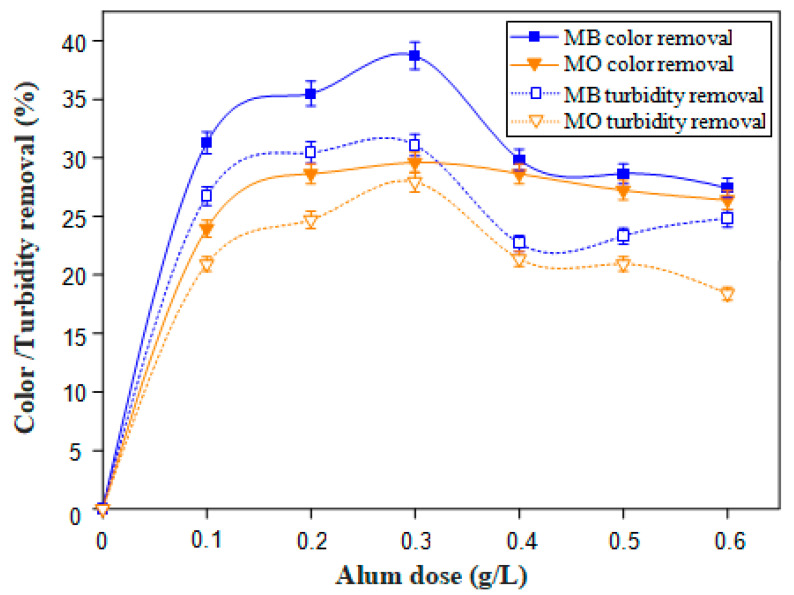
Color and turbidity removal for MB and MO solutions (C = 0.10 g/L) using only alum as coagulant.

The removal of the cationic dye, Methylene Blue (MB) and the anionic one, Methyl Orange (MO), was assessed via a coagulation-flocculation process using one of two cactus formulations (LP or DP) as a bio-flocculant in conjunction with aluminum sulfate (Al_2_(SO_4_)_3_·18H_2_O) as a coagulant under the initial dye solution conditions (Table 2). After preliminary series of coagulation assays using different alum doses (0.10–0.50 g/L), as shown in Figure 3, the highest color removal of 40 ± 1.1% for MB and 30 ± 0.8% for MO, as well as a turbidity reduction yield of nearly 30% for both dye solutions were achieved using only 0.30 g/L of alum.

Therefore, to shed light on the flocculating performance of the cactus formulations, a fixed optimum alum dose of 0.30 g/L was used for all subsequent flocculation experiments. It is worth recalling that results found by Beyene et al. [39] indicated an increase in the turbidity removal rate of a water sample from 23.9% to 54% and 28.5% to 58.2% as dose increased from 0.50 to 3.50 g for both cactus powder and alum used separately, respectively. The same authors also concluded that the combination of alum and cactus is more efficient for turbidity removal than either of them used separately. A combination of both of them was therefore adopted in this investigation.

**Figure 4:**
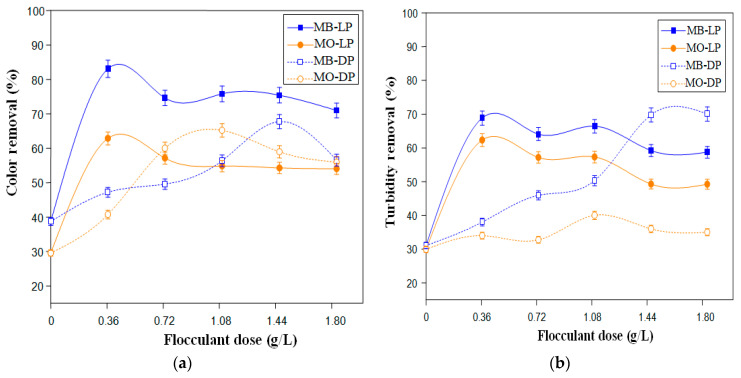
Color (**a**) and turbidity (**b**) removal for MB and MO using the two cactus powders (DP and LP) as flocculants (Initial MB and MO concentration = 0.10 g/L, coagulant dose = 0.30 g/L).

Figure 4 shows color and turbidity removal of the MB and MO synthetic solutions using the two cactus bio-flocculant powders (DP and LP) at different concentrations varying from 0.37 to 1.83 g/L (g/dry weight) added after an optimum dose of alum (0.30 g/L).

For both synthetic dye solutions (MB and MO), and as depicted in Figure 4a,b, color and turbidity removals rise with the increase of the cactus bio-flocculant doses until reaching an optimum dose where the highest color and turbidity abatements were observed. Figure 4a shows significant MB color removal efficiency of 83 ± 2.4% and 68 ± 2.0% using 0.37 g/L of the lyophilized cactus powder (LP) and 1.46 g/L of the dried cactus powder at 60 °C (DP), respectively. As well, almost the same trend was shown in Figure 4b for MB turbidity abatements under optimum doses for both cactus formulation-based flocculants. Like in the case of MB, dealing with the anionic dye, MO afforded color eliminations of 63 ± 1.8% and 65 ± 2.0% using 0.37 g/L of LP and 1.10 g/L of DP, respectively (Figure 4a). These optimal doses also provided a turbidity reduction of 62 ± 1.8% using LP and 37 ± 1.2% when it comes to DP (Figure 4b). Otherwise, it is worth noting that, for both synthetic tested dyes, the addition of cactus-based bio-flocculant beyond the optimal doses led to a slight pollutant removal efficacy decrease (Figure 4). Similar trend was pointed out by Amira et al. [55]. This feature can be ascribed to the re-stabilization of dyes molecules occurred by the dispersion of the insoluble cactus powders in the dye solutions. Therein, flocculants particles repel each other reducing subsequently color and turbidity removal. Furthermore, higher doses of cactus may also bring color formation due to the high chlorophyll content of cactus powder, consequently turbidity and color removal yield might decline.

The flocculating performance of LP and DP toward MB and MO color and turbidity abatement is in agreement with some published studies. For instance, in treating an industrial dyeing mesh effluent using lyophilized cactus powder, de Souza et al. [56] found a turbidity abatement around 85.4%. Further, to remediate a jeans laundry wastewater, a significant turbidity removal of 91% was achieved. Moreover, the flocculating effectiveness of the oven-dried cactus powder in terms of color and turbidity removal was in the order of 88.4% and 82.6%, respectively while dealing with a painting wastewater [57]. Additionally, in a study conducted by Kazi and Virupakshi [26] to treat a tannery effluent, a turbidity abatement of 78% was registered. Furthermore, in monitoring the turbidity elimination from river water using the oven-dried cactus powder, Al-Saati et al. [22] observed turbidity removal yields varying between 60% and 100%. In summary, taking into consideration these literature findings, salient turbidity and color removal have been attained using an oven-dried and chiefly the lyophilized cactus cladode-based flocculants.

To better select the effective cactus formulation which led to the highest color and turbidity removal performance, obtained results under the optimized doses of both formulations are summarized in Table 5.

Results listed in Table 5 indicate clearly that the highest color and turbidity removal were obtained with LP formula. For both cationic (MB) and anionic (MO) synthetic dye solutions, color and turbidity abatements were over 62%. Additionally, it is interesting to notice that the highest color (83 ± 2.4%) and turbidity (69 ± 2.0%) drop were achieved using a small dose of LP-based flocculant in the order of 0.37 g/L. These findings are in concordance with Noor et al. [58] survey stating that freeze-dried moringa, a concurrent plant-based flocculant for cactus, showed the most efficient removal power compared to the raw extract.

The superiority in the flocculation activity of the lyophilized cactus cladodes can be traced back to the high uronic acids concentration which consists mainly of polygalacturonic acid (Table 3). As well, the lyophilized cactus powder outperformed the oven-dried cladodes due to the lyophilization technique features, which are based on the dehydration of the sample, without damaging its bio-chemical composition, ensuring the stability of the material structure, as reported by Nireesha et al. [59]. In contrast, the oven-drying process may cause the distortion of the carbohydrate chains due to their decomposition, therefore affecting the availability of hydroxyl groups.

### 3.3. Effect of Storage Period on the Flocculation Activity of LP

With similarity to all biological materials derived from plants, cactus extract effectiveness might be affected over time. It is worth noting that Sellami et al. [30] reported that raw juice had lost significantly its flocculation power within seven days once kept at room temperature. Likewise, De Souza et al. [60] noticed that a storage period of four days at room temperature did not affect cactus extracts coagulation activity, however a drastic change occurred starting from the 5th day when a great loss in turbidity removal was registered. Almost the same conclusion was made by Dalvand et al. [61] while dealing with *Moringa oleifera* aqueous extract which was stable for 30 days. After that, an offensive odor was felt and its efficiency for dye removal was hampered. Elucidating the behavior and performance over time of the LP extract to remove dyes is hence crucial from the commercial standpoint. Therefore, the stability of the LP extract (exempt from water) was assessed for a period of 8 months with respect to MB removal capacity. Throughout this period, for every 15 days, a stored LP sample was taken out from the desiccator and tested for its flocculation activity, in the dual system with alum, by measuring the turbidity removal from the MB solution (C = 0.10 g/L). Based on the optimum conditions previously stated for MB removal, the flocculation tests were performed using 0.30 g/L of alum as coagulant in combination with an optimum dose of the stored LP in the order of 0.37 g/L.

**Figure 5:**
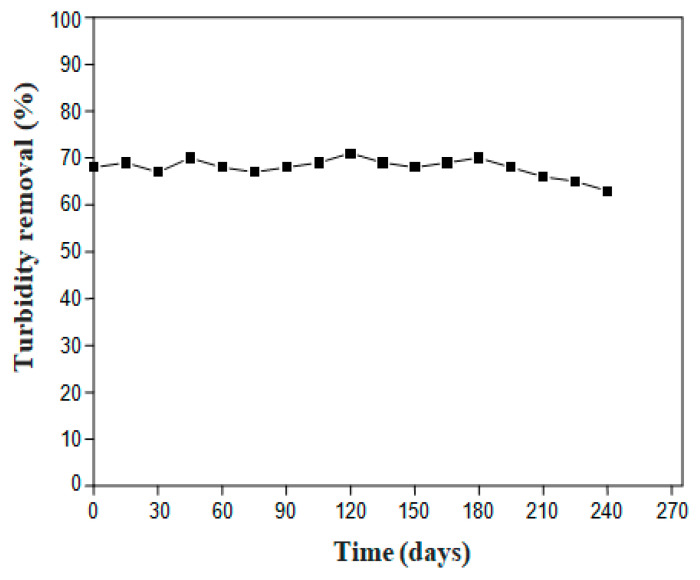
Effect of the LP storage period on the MB turbidity removal (MB concentration = 0.10 g/L, alum as coagulant: dose = 0.30 g/L, LP as flocculant: dose = 0.37 g/L).

Figure 5 depicts the removal power of the LP extract against time. It can be concluded that the flocculation proprieties did not vary significantly, at room temperature, under the storage conditions (hermetically sealed in plastic containers) and the period of preservation. Nonetheless, a small change in color (from a bright greenish to a fade greenish) was noticed and this might be resulting from the oxidation of chloroplast compounds in cactus pads. Bio-chemical composition and FTIR analysis should be carried out to see whether there is a change in LP components.

### 3.4. Plausible Mechanisms of Flocculation

A full grasp of the mechanisms involved in the flocculating activity of the lyophilized powder (LP) is of great interest for the ongoing scientific communities’ debate. As previously mentioned and reported elsewhere [47], carbohydrates (neutral sugars and uronic acids) are assumed to be the active flocculating agents in the cactus lyophilized powder. Among the significant amount of uronic acids in the LP formulation, polygalacturonic acid is reported to be the prevailing flocculating compound [54]. It consists of a long polymeric chain that entails high number of functional groups mainly carboxyl (-COO^−^/-COOH) and hydroxyl (-OH) groups. These functional groups provide available adsorption sites to positively charged pollutant molecules as well as to negatively charged ones with the latter preferentially by means of divalent cation (Ca^2+^) bridges, conferring to the cactus powders an amphoteric behavior that would enable them to eliminate or reduce the cationic and anionic dye particles at the same time.

**Figure 6:**
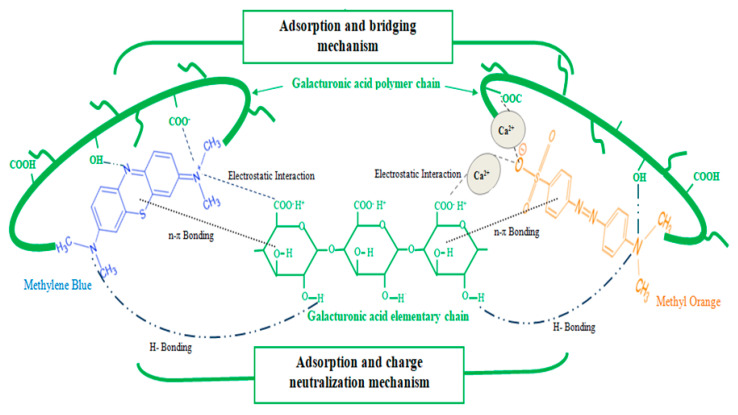
Illustration for the flocculation mechanisms with polygalacturonic acid and its different interactions with the dyes.

In this respect, Figure 6 draws a sketch of the presumed different chemical interactions between MB or MO and the main cactus flocculating agent; polygalacturonic acid.

As presented in Figure 6, the significant MB reduction can be explained merely by the high affinity of the negatively charged cactus compounds toward the cationic dye. In fact, an important electrostatic interaction between the positively charged MB molecules and the negatively charged polygalacturonic acid chain can take place and the charge neutralization phenomenon occurs. Moreover, the amino groups of MB may interact via hydrogen bond with the hydroxyl group or protonated carboxylic acid group of bio-flocculant. Further, along with charge neutralization, polygalacturonic acid, the presumed active compound for cactus extracts, comprises a long polymeric chain enabling cactus-based bio-flocculant to bridge the MB molecules with loops and tails. Therefore, it can be said that the adsorption-charge neutralization and bridging mechanisms occur concurrently in this case.

Whereas, in regard to MO dye, removal can be traced back to the amphoteric behavior of cactus extracts. In spite of the prevalent anionic character of the cactus polysaccharides due to the deprotonation of carboxylic acid groups (COO^−^ H^+^) in water, the presence of a considerable content of divalent cations (Ca^2+^) in the LP structure (Table 3) serves to neutralize the negative charges of the bio-flocculant and dye through the formation of bridges between the anionic long chains of polygalacturonic acid and the anionic MO molecules. On the other hand, the free electron pairs of nitrogen atoms in MO can establish hydrogen bonds with the hydroxyl groups of bio-flocculant, similarly to interactions with MB. n-π interactions between the polygalacturonic acid and the delocalized π orbital system of MO may also be involved. Taking into consideration the anionic surface charge of MO, the prevailing mechanism of flocculation is presumed to be adsorption and bridging. These features of removal performance are also well documented in the report of Debora et al. [62]. Likewise, Bouaouine et al. [63] suggested that, in dealing with anionic pollutants carrying the same charge as cactus active agents, flocculation does not operate through charge neutralization of pollutants, but instead mainly through an adsorption and bridging mechanism between particles.

### 3.5. Comparison of the Flocculating Ability of LP with the Well Known Chemical Flocculant (Polyacrylamide) and Some Other Bio-Based Flocculants

**Figure 7:**
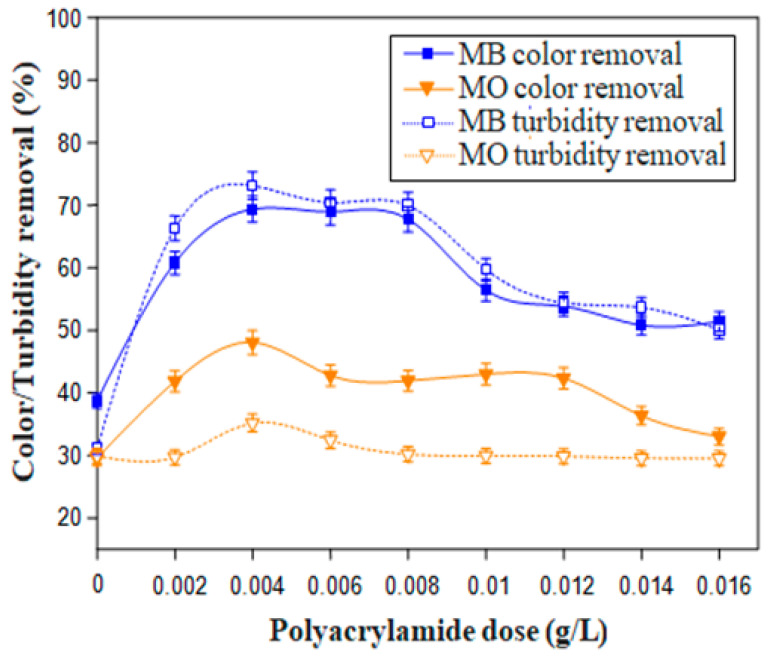
Flocculation performance of the chemical flocculant (polyacrylamide) (Dyes concentration = 0.10 g/L, coagulant dose = 0.30 g/L).

For the sake of a comparison between removal powers of the most efficient formulation (LP) and the most commonly used chemical flocculant known as polyacrylamide (Non-ionic polyacrylamide (PAM)), experiments of dyes removal were also carried out using this latter flocculant. Figure 7 displays the flocculation performance of PAM for different doses as flocculants (0.002–0.016 g/L) in conjunction with alum as coagulant (0.30 g/L). As depicted in Figure 7, the highest color and turbidity removal for both dyes (MB and MO) were registered for an optimum PAM dose of about 0.004 g/L.

**Figure 8:**
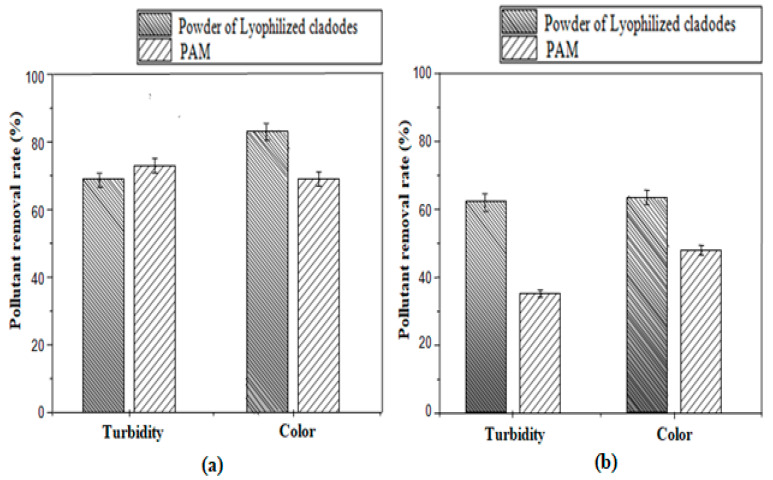
Turbidity and color removal using the natural flocculant (LP) and the chemical flocculant (polyacrylamide (PAM)) for MB (**a**) and MO (**b**) solutions with the optimized doses (Dyes concentration= 0.10 g/L, coagulant dose = 0.30 g/L, LP = 0.37 g/L, PAM = 0.004 g/L).

As well, based on the experimental flocculation tests, turbidity and color removal using the natural flocculant (LP) and the widely used synthetic PAM, are compared in Figure 8.

Figure 8a shows that in treating MB solution with PAM (0.004 g/L), significant turbidity and color reduction of 73.1 ± 2.2% and 69.3 ± 2.0% were respectively accomplished. Whereas, for the LP, although the graph registers almost the same turbidity drop, a slightly higher color abatement (83 ± 2.4%) with this latter bio-flocculant was achieved. As well, in case of MO solution (Figure 8b), it can be noticed that, using PAM, turbidity and color removals were 35.2 ± 1.4 and 48.0 ± 2.0%, respectively, against 62 ± 1.8% and 63 ± 1.8% for the LP. Therefore, according to these flocculation tests, LP seems to be a promising candidate to the chemical flocculant PAM, although a higher bio-flocculant dose is required. In fact, as the cactus cladodes are widely available in some regions, valorization of this natural residue as a flocculant has an additional positive environmental impact owing to its bio-degradability and promotes their application instead of the synthetic chemical flocculants.

On the other hand, to assert the flocculating power of LP, a comparison with other bio-materials widely used, was carried out. Table 6 shows pollutant removal rates using various bio-based flocculants for different dye-bearing wastewaters remediation. Experimental findings listed in Table 6 point out that lyophilized cactus cladodes powder (LP) has comparable flocculating activity to other bio-flocculants. Color and turbidity removal yields from the different dyes-bearing effluents are around 80 and 60%, respectively (Table 6). As well, it is worth noting that lyophilized cactus cladode-based flocculant achieved higher color and turbidity abatement of 83 ± 2.4% and 69 ± 2.0%, respectively over the other natural flocculants such as moringa seeds (70%) [64]. Hence, lyophilized cactus cladodes could serve as an outstanding local, effective, and eco-friendly flocculant specially in countries where the plant grows and can be easily cultivated such as in case of most north African countries. It is also worth referring that the cactus is a drought-resistant plant that can cope well with climate change affecting most of the world countries, and therefore the sustainability in terms of supply would be guaranteed.

## 4. Conclusions

The efficacy of cactus cladodes as a bio-flocculant under two formulations, namely lyophilized (LP) and oven-dried at 60 °C (DP) powders, was evaluated toward the removal of two popular cationic (Methylene Blue) and anionic (Methyl Orange) dyes from synthetic solutions via adding the cactus cladodes powders after a coagulation stage. Significant color and turbidity abatements were accomplished with a more pronounced removal yield for both dye solutions when the LP formula was used. The effect of the storage period and conditions on the shelf life of this latter formula was assessed for eight months via turbidity removal and it could be concluded that it was highly insignificant.

The bio-chemical characterization carried out indicated a significant load of neutral sugars, uronic acids and calcium, presumed to be the flocculation active agents. Additionally, the FTIR spectra for both powders corroborated the prevalence of the polysaccharide components, by showing characteristic peaks of, hydroxyl, carboxyl, methoxy and other functional groups.

Due to their considerable amount in the LP, carbohydrates, chiefly uronic acids such as polygalacturonic acid, are supposed to be the main flocculating agents leading to dye flocculation through adsorption/charge neutralization and bridging mechanisms.

The significant color and turbidity decline of the treated dye solutions promote the application of natural, safe and eco-friendly flocculants like the lyophilized cactus cladodes as an alternative to the conventional synthetic ones, namely polyacrylamide. Considering their efficiency, availability, the easiness of their cultivation and their stability over time, cactus cladode-based natural flocculants such as the LP could be a feasible approach to mitigate dye threats and the intensive industrial chemicals upshot to wastewaters.

## Figures and Tables

**Table 1:** Analytical methods for bio-chemical components extraction and quantification.

Bio-Chemical Content	Protocol of Extraction	Reference
Total phenols	In a test tube, 5 mL of Folin-Ciocalteu reagent was mixed with 1 mL of cactus extract which was previously prepared by adding 2 g of powder to 20 mL of methanol. Thereafter, 4 mL of sodium carbonate solution (7.5%) was added to the mixture. The absorbance of the final solution was assessed at 760 nm using gallic acid as a standard reagent.	[31]
Proteins	1 g of cactus powder was added to 20 mL of distilled water. 0.4 mL of this suspension was mixed with 4 mL of an analytical reagent previously prepared. Then, 0.4 mL of Folin-Ciocalteu reagent was added to the mixture. The analytical reagent was prepared by mixing 2 mL of solution 1, (CuSO_4_ (1.56 %, *w*/*v*) and sodium potassium tartrate (2.37 %, *w*/*v*) with 100 mL of solution 2 (Na_2_CO_3_ (2 %, *w*/*v*) and NaOH (0.4 % *w*/*v*). Proteins were determined by absorbance measurement at 660 nm using Bovine Serum Albumin as a standard solution.	[32]
Neutral Sugars	A cactus solution was prepared by mixing 0.5 g of cactus powder with 20 mL of concentrated sulfuric acid and adjusting with distilled water to 500 mL. From this solution, 2 mL was mixed with 1 mL of phenol (5%) followed by a supplement of 5 mL of sulfuric acid (96%). For neutral sugars estimation, glucose was used as standard solution and absorbance was measured at 490 nm.	[33]
Uronic Acids	To 1 mL of cactus sample prepared via an acidic hydrolysis of 50 mg of cactus powder, 6 mL of sulfuric acid solution containing 0.0125 M sodium tetraborate was added. Then, a supplement of 200 µL of m-hydroxybiphenyl was added to the mixture. Absorbance was registered at 520 nm and quantified based on galacturonic acid as a standard reagent.	[34]
Klason Lignin	0.9 g of cactus powder was mixed with 9 mL of H_2_SO_4_ (72%). The mixture was placed into an autoclave at 121 °C for 1h. Thereafter, the suspension was filtered after settling overnight to acquire the klason lignin fraction which is the insoluble lignin obtained by oven-drying the filtration residue. The residue was then weighed using an accurate balance.	[35]

**Table 2:** Physico-chemical characteristics of methylene blue (MB) and methyl orange (MO).

Dye	pH *	λ (nm) *	Turbidity (NTU) *	Structure
MB	7.2	663	95.6	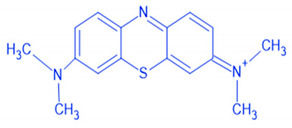
MO	6.2	464	80.2	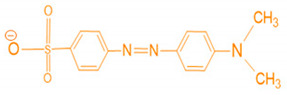

* for 0.1 g/L aqueous solution.

**Table 3:** Bio-chemical compositions of the cactus formulations.

Composition (mg/g DW)	LP	DP
Total phenols	2.3	2.0
Proteins	54	64
Neutral sugars	345	380
Uronic acids	682	546
Klason lignin	15.7	25.6
Calcium (Ca)	72.4	79.5
Sodium (Na)	1.5	2.3
Magnesium (Mg)	7.1	11.6
Iron (Fe)	0.035	0.075

LP: Lyophilized Powder; DP: Dried Powder at 60 °C; DW: Dry Weight.

**Table 4:** Main functional groups in the FTIR spectra of cactus cladodes powders.

Wave Number (cm^−1^)	Functional Groups in Polysaccharides (Vibration Type)
3355	O–H (stretching)
2931	C–H (stretching)
~1730	Non-ionized COOH/substituent methyl ester (C=O stretching)
1623	Ionized COOH (COO^−^ asymmetric stretching)
1425	Ionized COOH (COO^−^ symmetric stretching)
1046	Alcohol/ether (C–O stretching)

**Table 5:** MB and MO color and turbidity removals using cactus formulations as flocculants after a coagulation stage.

Synthetic Dye Solution (0.10 g/L)	MB		MO	
Cactus formulation	LP	DP	LP	DP
Optimum dose (g/L)	0.37	1.46	0.37	1.10
Color removal (%)	83 ± 2.4	68 ± 2.0	63 ± 1.8	65 ± 2.0
Turbidity removal (%)	69 ± 2.0	70 ± 2.0	62 ± 1.8	37 ± 1.2

**Table 6:** Flocculating ability of various bio-materials.

Natural Coagulants/Flocculants	Treated Waters	Optimum Dose (g/L)	Color Removal (%)	Turbidity Removal (%)	References
Chitosan	Acid Blue 292 dye	0.003	52	n.d	[65]
Fique leaves	Textile industry	0.01	89	93	[66]
Cicer arietinum seeds	Congo red dye	0.80	89	n.d	[67]
Moringa seeds	Laundry wastewater	0.12	n.d	83	[68]
Cassava peels	Institutional wastewater	0.40	n.d	81	[69]
Moringa seeds	Methylene blue dye	50	70	n.d	[64]
Lyophilized cactus cladodes	Methylene blue dye	0.37	83	69	This study
Lyophilized cactus cladodes	Methyl Orange	0.37	65	62	This study

n.d: not determined.
